# Long non‐coding RNA AK085865 ablation confers susceptibility to viral myocarditis by regulating macrophage polarization

**DOI:** 10.1111/jcmm.15210

**Published:** 2020-03-27

**Authors:** Yingying Zhang, Xueqin Li, Xiang Kong, Mengying Zhang, Deguo Wang, Yinhua Liu, Kun Lv

**Affiliations:** ^1^ Key Laboratory of Non‐coding RNA Transformation Research of Anhui Higher Education Institutes (Wannan Medical College) Wuhu China; ^2^ Department of Laboratory Medicine The First Affiliated Hospital of Wannan Medical College Wuhu China; ^3^ Central Laboratory The First Affiliated Hospital of Wannan Medical College Wuhu China; ^4^ Department of Gerontology The First Affiliated Hospital of Wannan Medical College Wuhu China; ^5^ Department of Pathology The First Affiliated Hospital of Wannan Medical College Wuhu China

**Keywords:** AK085865, long non‐coding RNA, macrophage polarization, viral myocarditis

## Abstract

Accumulating evidence indicates that regulators of macrophage polarization may exert pivotal functions in the development of coxsackievirus B3 (CVB3)‐induced viral myocarditis (VM). However, the mechanisms underlying macrophage polarization remain to be explored. Here, we sought to identify novel and functionally important long non‐coding RNAs (lncRNAs) during macrophage polarization and to investigate their function and contribution to VM. In this study, we identified the lncRNA AK085865 as an important regulator of macrophage polarization. Knock‐down of AK085865 diminished phenotypical expression of M2 macrophages while promoting polarization to the M1 phenotype. Moreover, AK085865^−/−^ mice had increased susceptibility to CVB3‐induced VM. We observed striking bias towards M1 macrophages, whereas the M2 population was decreased in AK085865^−/−^ VM mice. Collectively, our findings uncover a critical role of AK085865 in the regulation of macrophage polarization in vitro and in vivo, identifying a new player in the development of VM and providing a potential clinically significant therapeutic target.

## 1 INTRODUCTION

Viral myocarditis (VM), characterized by severe cardiac inflammation, is a major cause of both congestive heart failure and sudden cardiac death in healthy young persons. Similar to infected patients, mice infected with coxsackievirus B3 (CVB3) cause acute viral myocarditis on days 7 to 14 post‐infection and progresses to autoimmune myocarditis and dilated cardiomyopathy (DCM) on the 35th day after infection.^1^, ^2^, ^3^ Despite decades of abundant research work, the pathogenesis of VM remains undefined.

Although CVB3‐induced VM is thought to be a CD4^+^ T lymphocyte–mediated inflammatory heart disease,^4^, ^5^ accumulated data suggest that macrophages are the major inflammatory infiltrating cells, which play a role in the development of VM. Macrophages, as major regulators of inflammation, possess highly plasticity and heterogeneous. Mirroring the Th1/Th2 nomenclature, macrophages can be divided into classically activated (M1) or alternatively activated (M2) macrophages depending on distinct microenvironmental stimuli.^6^, ^7^ M1 macrophages, have long been known to be induced by lipopolysaccharide (LPS) and interferon‐γ (IFN‐γ), typically produce large amounts of nitric oxide (NO) and pro‐inflammatory cytokines (interleukin [IL]‐12, tumour necrosis factor [TNF]‐α). In contrast, M2 macrophages are induced by Th2‐producing IL‐4 and IL‐13, with high secretion levels of anti‐inflammatory cytokines (IL‐10), and are characterized by increased arginase 1 (Arg1) activity, chitinase 3‐like 3 (Chi3l3 or YM‐1) and resistin‐like‐α (Retnlα or Fizz1).

Functionally, M1 macrophages represent pro‐inflammatory activities that are essential for host defence, and M2 macrophages are participated in tissue repair and homeostasis.^8^, ^9^ Previous studies have reported that macrophage‐depleted mice fail to develop VM, and the severity of myocardial inflammation is related to the intensity of the macrophage infiltration.^10^, ^11^ However, extensive macrophage infiltration is not always a manifestation of severe myocarditis. Frisancho‐Kiss et al^3^ and Huber et al^5^ observed that female BALB/c mice had significant macrophage infiltrate after CVB3 infection, and these mice were less susceptible to VM than other strains of mice. Furthermore, Li et al^12^ found that adoptive transfer of ex vivo‐programmed M1 macrophages showed a significant increase in myocarditis as expected, whereas transfer of M2 macrophages remarkably alleviated myocardial inflammation in susceptible male mice. Our previous studies have shown that microRNA‐155 confers susceptibility to VM by regulating macrophage polarization.^13^ Therefore, it can be speculated that macrophage polarization regulators may also play a key role in regulating cardiac inflammation. However, despite this process is important for VM, the mechanism of macrophage polarization remains to be explored.

Long non‐coding RNAs (lncRNAs) are RNA molecules that are longer than 200 bases and do not encode proteins.^14^ These RNAs can be either intronic, natural anti‐sense transcripts (NATs), intergenic or promoters.^14^ LncRNAs control gene transcription through combined with transcription factors, heterogeneous nuclear ribonucleoproteins (hnRNPs) or chromatin‐modifying factors. In addition, lncRNAs functions through post‐transcriptional mechanisms targeting the translation of host mRNAs, splicing or stability.^15^ Although lncRNAs have been found in almost all immune cells, their function in these cells is just beginning to appear.^16^, ^17^ As reported, lincRNA‐Cox2 induced by TLR ligands was identified as a dynamically regulated gene. It can in turn act both to repress and to promote inflammatory gene expression.^18^ In T cells, lncRNA NeST regulates the transcription of IFN‐γ gene and persistent infection with Theiler's virus,^19^ while Rmrp regulates the effector function of T helper 17 cells.^20^ Several additional lncRNAs, including THRIL, lnc13 and an anti‐sense lncRNA, AS‐IL‐1a, also regulate inflammatory gene expression in myeloid cells.^21^, ^22^, ^23^, ^24^, ^25^

In this study, we define the lncRNA AK085865 as an important regulator of macrophage polarization in vitro and in vivo. We found that AK085865 up‐regulated in M2 macrophages than in M1 macrophages. Knock‐down of AK085865 diminished M2 phenotypical expression while promoting polarization to the M1 phenotype. We also show here that AK085865^−/−^ mice had increased susceptibility to CVB3‐induced VM. We observed striking bias towards M1 macrophages, whereas the M2 population was decreased in AK085865^−/−^ VM mice. Taken together, our data lead us to conclude that AK085865 is an essential controller of macrophage polarization, regulating inflammatory responses.

## 2 MATERIALS AND METHODS

### 2.1 Ethics statement

All of the animal experimental procedures were approved by the Animal Ethics Committee of Yijishan Hospital (the approval number: No. AECYJS‐2016002) and were performed according to the Guide for the Care and Use of Laboratory Animals published by the US National Institutes of Health (8th Edition, National Research Council, 2011).

### 2.2 Mice

LncRNA AK085865 knockout (KO) mice on a C57BL/6 background were generated by the Nanjing Biomedical Research Institute of Nanjing University (Nanjing, China) and were housed in pathogen‐free mouse colonies. In brief, Cas9 mRNA and gRNA were obtained by in vitro transcription, and then, they were injected into fertilized eggs of C57BL/6 mice via the microinjection technique to generate F0 generation mice. The F0 generation mice with the gene mutant were backcrossed to C57BL/6 mice to obtain heterozygous F1 mice. The AK085865 KO (AK085865^–/–^) mice were generated by crossing heterozygous F1 mice. We determined the genotype of the mice by PCR analysis of genomic DNA isolated from mouse tails using the primers in Table S1.

### 2.3 Isolation and cultivation of murine BMDMs

Bone marrow cells were obtained by flushing the femurs from mice with Dulbecco's modified Eagle's medium (DMEM)‐HEPES medium (Gibco). Cells were collected in 50‐mL tubes and centrifuged for 10 minutes (100 *g*). The supernatant was removed, and cells were suspended in DMEM (20% FBS; 20% L929 supernatant). Then, 1 × 10^6^ cells were cultured in 6‐well plate at 37°C and 5% CO2 for 7 days (BMDMs). Macrophage polarization was obtained by removing the culture medium and culturing cells for an additional 48 hours in DMEM supplemented with 10% FBS and 100 ng/mL LPS (Sigma) plus 20 ng/mL IFN‐γ (PeproTech) (for M1 polarization) or 20 ng/mL IL‐4 (PeproTech) (for M2 polarization).

### 2.4 Microarray analysis

The microarray work was performed by Shanghai Biotechnology Corporation, Shanghai, China. In brief, total RNA from each sample was amplified and labelled by Low Input Quick Amp WT Labeling Kit (Agilent Technologies), following the manufacturer's instructions. The labelled cRNA was purified by RNeasy Mini Kit (Qiagen). The concentration and specific activity of the labelled cRNAs (pmol Cy3/μg cRNA) were measured by NanoDrop 2000. Each microarray slide was hybridized with 1.65 μg Cy3‐labelled cRNA using Gene Expression Hybridization Kit in Hybridization Oven, according to the manufacturer's instructions. After 17‐hour hybridization, slides were washed in staining dishes using the Gene Expression Wash Buffer Kit following the manufacturer's instructions. Next, the slides were scanned by Agilent Microarray Scanner G2565C (Agilent Technologies) with default settings, Dye channel: Green, Scan resolution = 3 μm, PMT 100%, 20 bit. Agilent Feature Extraction software (version 10.7) was used to analyse the acquired array images. Quantile normalization and subsequent data processing were performed using the GeneSpring Software 11.0 (Agilent Technologies). Differentially expressed genes (DEGs) were identified through fold change (greater than 2‐fold) filtering and with FDR adjusted *P* values < .05.

### 2.5 RT‐qPCR

Total RNAs were isolated with RNeasy Kits (QIAGEN), and cDNA was made using an iScript cDNA Synthesis Kit. SYBR Green dye‐based quantitative real‐time PCR was performed using the SYBR Green PCR Master Mix and CFX96 Real‐Time PCR System from Bio‐Rad. For miRNA real‐time PCR, a commercial Hairpin‐itTM miRNAs qPCR Quantification Kit (RiboBio) was used. Briefly, 2 μg RNA was used as template and then reverse‐transcribed using a miRNA‐specific RT‐primer. The resulting cDNA was further amplified with a universal reverse primer and a specific forward primer. Calculations of mRNA or miRNA expression levels were performed using the comparative CT (△△CT) method and normalized against *GAPDH* or *U6* snRNA levels. All reactions were run in triplicate. The primers used are shown in Table S1.

### 2.6 Small Interfering RNA and Transfection of BMDMs

LncRNA Smart Silencer (an siRNA mix containing three ASOs and three siRNAs, are shown in Table S2) targeting AK085865 at different sites and negative control (NC) with no definite target were synthesized by RiboBio (Guangzhou, China). BMDMs were seeded on six‐well plates at a density of 5 × 10^5^ cells/well overnight and then transfected with Smart Silencer or the negative control at a final concentration of 100 nmol/L using Lipofectamine 3000 (Invitrogen). The interfering efficiency was detected by RT‐qPCR after transfection, and the Smart Silencer with silencing efficacy of more than 70% was selected for further experiments.

### 2.7 RNA FISH and protein immunofluorescence

Cells grown on glass coverslips or tissue slices were fixed in 4% paraformaldehyde (PFA) for 30 minutes and permeabilized with 0.5% Triton X‐100. Then, cells were washed three times with phosphate buffer saline (PBS) and treated with pre‐hybridization buffer (2 × saline sodium citrate, 10% formamide). The FISH (Fluorescence in situ hybridization) probes to AK085865 (Exiqon) were resuspended in hybridization buffer (2 × saline sodium citrate, 10% formamide, 10% dextran sulphate) to a final concentration of 250 nmol/L per probe set. Hybridization was carried out in a humidified chamber at 37°C for 16 hours. After incubation, cells were washed three times with 4 × saline sodium citrate (SSC) plus with 0.1% Tween‐20 at 42°C, following with once 2 × SSC and once 1 × SSC at 42°C, and then washed once with PBS at room temperature. For immunofluorescence staining experiment, cells were fixed in 4% paraformaldehyde (PFA) for 30 minutes and permeabilized with 0.25% Triton X‐100. Then, cells were washed three times with PBS and treated with the first antibody at 37°C; 2 hours later, cells were washed three times with PBS and treated with fluorescent‐labelled secondary antibody for 1 hour at 37°C. Nuclei were labelled with DAPI. We continued with several rounds of washing and finished with mounting the coverslip onto a microscope slide using an anti‐fade mounting medium. All immunofluorescence staining was photographed under a confocal microscope. The information about antibodies used is as follows: CD68 antibody (diluted at 1:200, Abcam), iNOS antibody (diluted at 1:100, Abcam), Arginase antibody (diluted at 1:1000, Abcam) and Goat anti‐Mouse IgG (ab150115, Abcam).

### 2.8 3′ RACE and 5′ RACE

The RNA‐ligase‐mediated RACE (RLM‐RACE) was carried out with total RNA extracted from primary myoblasts culture and was used to determine the transcription start points and the size of the AK085865 transcripts. Rapid amplification of 5′ or 3′ cDNA ends was carried out using a Smart RACE cDNA Amplification Kit (Clontech), according to the manufacturer's instructions. Primers used are as follows: 5′ RACE (AAGCAACTATGGAGATATGATTAAC); 3′ RACE (CCAAGCAAGACAGCCAGGCAGCTA).

### 2.9 Virus and myocarditis model

The original stock of CVB3 (Nancy strain) was a gift from Professor Wei Hou (School of Basic Medical Sciences, Wuhan University) and was maintained by passage through HeLa cells (ATCC number: CCL‐2). Virus titre was routinely determined prior to infection by a 50% tissue culture infectious dose (TCID_50_) assay of HeLa cell monolayers according to previously published procedures.^26^ Mice were infected by an intraperitoneal (ip) injection of 0.1 mL of phosphate buffered saline (PBS) containing approximately 1 × 10^5^ plaque‐forming units (PFU) of the virus on day 0. Tissue or cells were collected on day 7.

### 2.10 Generation of bone marrow chimeric mice

One week before irradiation, feed the recipient mice with medicated water. Bone marrow cells were prepared from WT or AK085865 KO donor mice and adoptively transferred into lethally irradiated (950 rad, in two divided doses) AK085865 KO or WT recipient mice (8‐week‐old, 1 × 10^7^ per mouse). After transplantation, the recipient mice were continued to give medicated water for 1 week. After 8 weeks, the chimeric mice were subjected for myocarditis model induction.

### 2.11 Histopathology

Hearts were cut longitudinally and fixed in 10% phosphate buffered formalin and embedded in paraffin. Sections (5 μm thick) were cut at various depths in the tissue section and stained with haematoxylin and eosin (H&E) to determine the level of inflammation. Sections were examined by two independent investigators blinded to the disease state of the mice.

### 2.12 Cytokines ELISA

Cytokine levels of TNF‐α, IL‐12, IFN‐γ, IL‐4 and IL‐13 were measured in homogenized heart or cell supernatants using respective cytokine ELISA kits (R&D Systems), according to the manufacturer's instructions.

### 2.13 Plaque assay

Viral titres were determined by plaque assay as described previously.^27^ Briefly, HeLa cells were seeded into six‐well plates (8 × 10^5^/well) and incubated at 37°C. Cell monolayers at ~90% confluence were washed with PBS and then overlaid with 500 μL of serial 10‐fold dilutions of supernatants from heart homogenates. The cells were incubated for 1 hour, and the supernatants removed. The cells were then overlaid with 2 mL of sterilized soft agar, incubated at 37°C for 72 hours, fixed with Carnoy's fixative (ethanol: acetic acid = 3:1), for 30 minutes, and stained with 1% crystal violet. The plaques were counted, and the numbers of viral PFU/mL were calculated.

### 2.14 FACS analysis

Single‐cell suspensions were pooled from heart tissues. Surface markers were stained with fluorochrome‐conjugated mAbs diluted in 1% FBS in PBS: CD4, CD62L, F4/80, Arg1 and iNOS (eBioscience; BD Pharmingen; Biolegend). For intracellular staining, cells were fixed and permeabilized using fixation buffer and permeabilization solution (eBioscience). Cell fluorescence was measured using FACS (Beckman Coulter), and data were analysed using FlowJo software (Treestar).

### 2.15 In vivo proliferation assay

The proliferation of CD4^+^ T cells was determined by 5‐bromo‐2‐deoxyuridine (BrdU) incorporation assay in vivo. BrdU (Sigma) was supplied with an injection (1 mg in 1 mL PBS) 1 day before termination of mice. The heart infiltrated cells were isolated and stained with conjugated anti‐mouse CD4 antibody (eBioscience), fixed in IC fixation buffer (eBioscience) for 30 minutes and permeabilized in permeabilization buffer (eBioscience) for 20 minutes. Cells were then incubated at RT for 30 minutes in 0.15 mol/L NaCl, 4.2 mmol/L MgCl_2_ and 10 mmol/L HCl in the presence of 2 U DNaseI (Invitrogen), followed by staining with conjugated anti‐BrdU antibody (BD Biosciences) for 30 minutes, and were finally analysed by FACS (Beckman Coulter).

### 2.16 Griess assay

Tissue homogenate of myocardial‐infiltrating macrophages in lncRNA AK085865^−/−^ and WT mice during CVB3‐induced VM was prepared, and then, 50 μL aliquots of the tissue homogenate were mixed with 50 μL of Griess reagent (Beyotime Biotechnology, China) and incubated for 10 minutes at room temperature in the dark. The colorimetric reaction was then measured at 540 nm using a Multiskan Go microplate reader (Thermo Scientific).

### 2.17 Arginase activity assay

Myocardial‐infiltrating macrophages in lncRNA AK085865^−/−^ and WT mice during CVB3‐induced VM were prepared. Then, 1 × 10^6^ macrophages were lysed with 50 μL of 0.1% Triton X‐100 for 30 minutes and then added to 50 μL of 50 mmol/L Tris‐HCl/10 mmol/L Cl_2_Mn·4H_2_O (pH 7.5) and incubated at 55°C for 10 minutes. L‐arginine hydrolysis was carried out by incubating with 25 μL of 0.5 mol/L L‐arginine (pH 9.7) at 37°C for 60 minutes. The reaction was then stopped with 400 μL of stop solution (H_2_SO_4_ (96%)/H_3_PO_4_ (85%)/H_2_O (1:3:7, v/v/v)) and 25 μL of 9% of 2‐isonitrosopropiophenone. The reactions were incubated at 100°C for 45 minutes, and 100 μL of each sample was analysed using a microplate reader at 540 nm.

### 2.18 Hemodynamics analysis

After 7 days following CVB3 injection, the mice were anesthetized with intraperitoneal injection of 1% pentobarbital sodium (50 mg/kg). The mice were endotracheally intubated and mechanically ventilated with room air (respiratory rate 45 breaths/min, respiration ratio 1:1, tidal volume 1.5 mL). The left ventricular (LV) performance was assessed using a self‐made polyethylene catheter inserted via the right carotid artery as previously described.^28^ Briefly, the catheter (0.3 mm × 0.6 mm ID/OD) which prefilled with fresh heparinized saline (500 U/mL) was inserted into the LV through cardiac apex. The catheter connected to a pressure transducer which linked with a biological data acquisition system (RM6240, Chengdu, China). After stabilization, the readout signals were stored and displayed in a computer to determine off‐line LV end‐diastolic pressure (LVEDP), LV systolic pressure (LVSP), the maximum first derivative of LV pressure (+d*P*/d*t*_max_) and minimum first derivative of LV pressure (−d*P*/d*t*_min_).

### 2.19 Statistical analysis

Data are shown as the mean ± SD. Statistical analysis of the data was performed with the two‐tailed independent Student's *t* test and ANOVA analysis using SPSS, and the Kaplan‐Meier survival curves were determined using GraphPad Prism v5.0 (GraphPad Software, Inc). *P* < .05 was considered statistically significant.

## 3 RESULTS

### 3.1 Identification of lncRNAs in ex vivo‐programmed M1 and M2 macrophages

**FIGURE 1:**
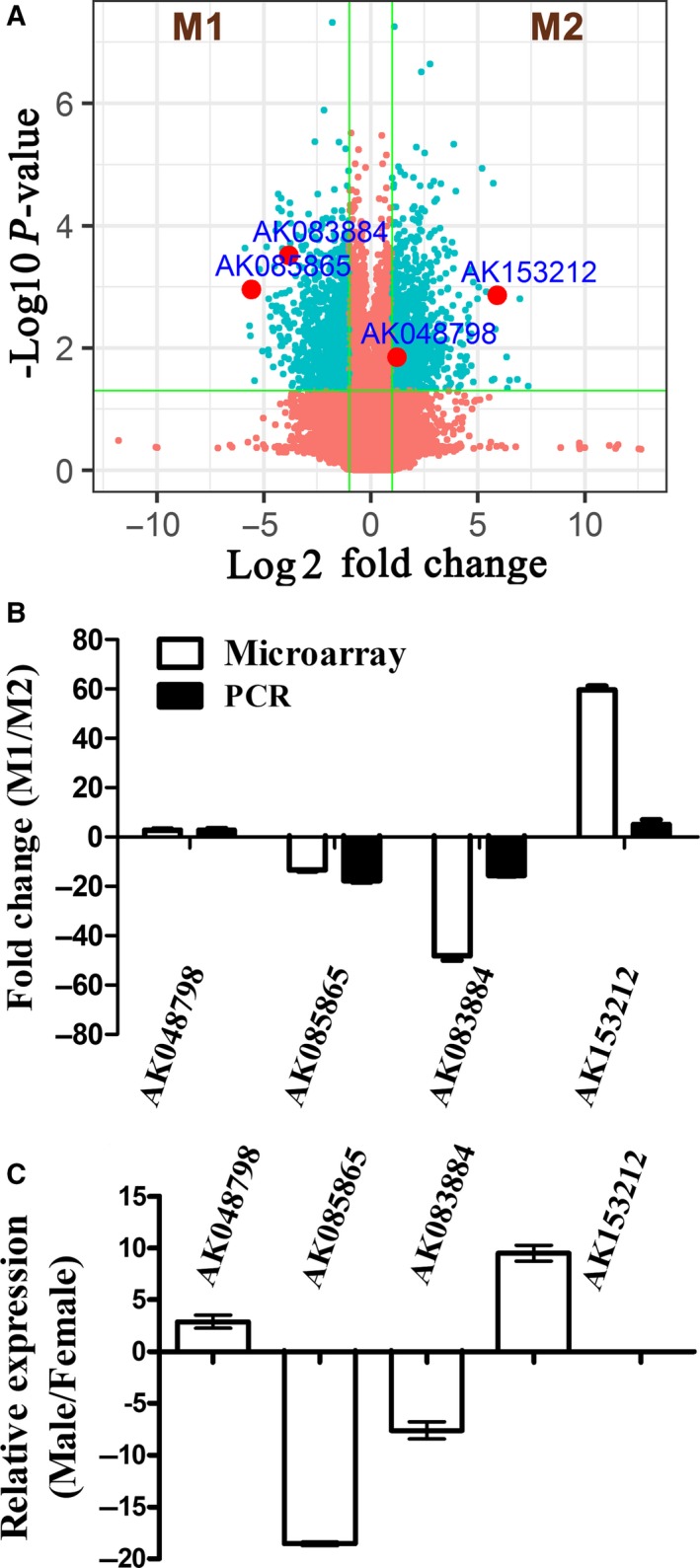
Identification of lncRNAs in regulating macrophage polarization. BMDMs were stimulated with LPS (100 ng/mL) and IFN‐γ (20 ng/mL) or IL‐4 (20 ng/mL) for 48 h to establish M1 and M2 macrophages. A, Volcano plots showing the differences in lncRNA expression between the M1 and M2 macrophages. The values of the x‐ and y‐axes in the scatter plot are averaged normalized values in each group [log2 fold changes (*x*‐axis) vs ‐log10 *P*‐value (*y*‐axis)]. B, Validation of selected lncRNAs differentially expressed in polarized macrophages. C, Expression of selected lncRNAs in myocardial‐infiltrating macrophages from male and female mice during VM (n = 6‐8/group). Data were expressed as the means ± SD of three independent experiments

Microarray analysis was performed in in vitro‐polarized M1 and M2 macrophages to screen the differentially expressed lncRNAs in macrophage polarization. Among the 33,231 lncRNAs identified, there were 627 lncRNAs that were enriched in M1 macrophages and 624 lncRNAs that were enriched in M2 macrophages with the selection criteria of >2‐fold differentially expressed changes and with FDR adjusted *P* values < .05 (Figure 1A).

According to the neighbour gene function of differentially expressed lncRNAs, we selected a group of lncRNAs and validated the results by RT‐qPCR to validate the results of the lncRNA microarray analysis. The 4 lncRNAs (AK048798, AK085865, AK083884 and AK153212), selected on the microarray as being the most differentially expressed in M1 as compared to M2 macrophages, were indeed confirmed by RT‐qPCR analysis to be differentially expressed (Figure 1B). Furthermore, studies have revealed that the phenotypes of myocardial‐infiltrating macrophages in male and female mice at the acute phase of VM were different. Males differentiate into M1 type, while females are mainly M2 type.^12^ We next analysed the expression of the above four lncRNAs in heart‐infiltrating macrophages. LncRNA AK085865 was the most prominent and abundant molecule in the heart‐infiltrating macrophages of female mice compared to male mice at the acute phase of VM (Figure 1C).

### 3.2 Characterization analysis of the lncRNA AK085865 sequence and expression pattern

**FIGURE 2:**
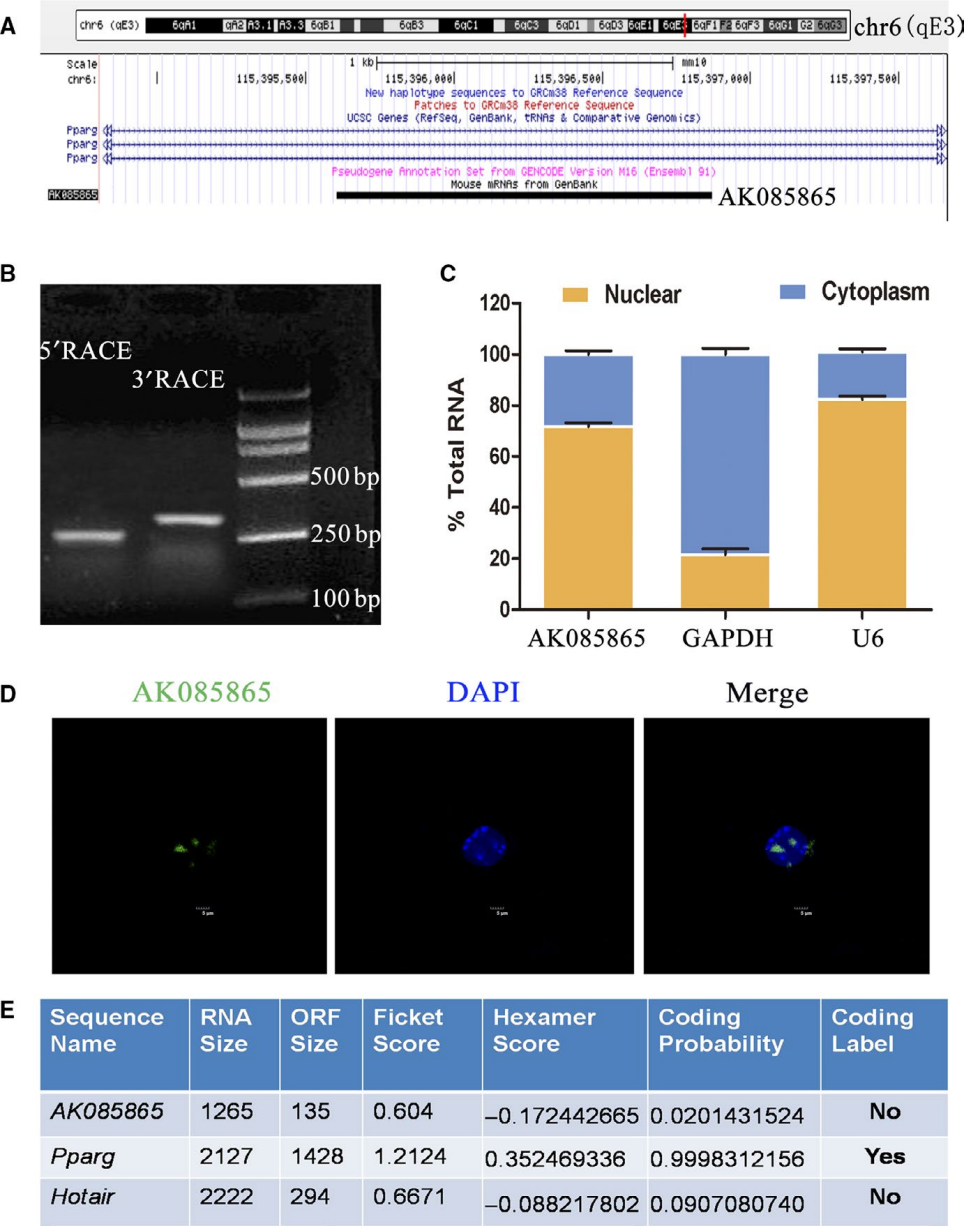
Characterization of lncRNA AK085865. A, AK085865 locates at the second intron of PPARγ gene on mouse chromosome 6. B, Results of 5′ RACE and 3′ RACE of AK085865 in M2. C, RT‐qPCR analysis of RNA extracted from nucleus (yellow) and cytosol (blue) in BMDMs. D, RNA FISH detecting endogenous AK085865 molecules (green) in resting BMDMs. DNA (blue) was stained with DAPI. A representative image (Scale bar = 5 μm) is shown. E, AK085865 was predicted to be a non‐coding RNAs. The RNA sequences of AK085865, PPARγ and HOTAIR were put into the CPAT program, and both AK085865 and HOTAIR were predicted to be non‐coding RNAs, while PPARγ RNA was identified to code for protein

Since many lncRNAs have been shown to have positive or negative regulatory effects on their neighbour genes,^29^, ^30^, ^31^ the genomic locations of these neighbouring genes need further analysis. AK085865 is located on chromosome 6 in mice and transcribed from the second intron of protein‐coding genes PPARγ (Figure 2A). It has been reported that PPARγ could modulate the response of macrophages to M1 or M2 stimulus,^32^ and we next investigated whether AK085865 regulates the expression of PPARγ transcript. RT‐qPCR and Western blot results showed that transfection of primary BMDM cells with AK085865 Smart Silencer resulted in a statistically significant knock‐down of PPARγ mRNA and protein levels (Figure S1). Using 5′ Rapid Amplification of cDNA Ends (RACE) and 3′ RACE, we determined that AK085865 was 1266 base pairs (bp) length (Figure 2B). Cell fractionation followed by RT‐qPCR analysis showed that about 70% of the AK085865 transcripts were located in the nucleus (Figure 2C). Fluorescence in situ hybridization (FISH) also demonstrated that AK085865 was mainly enriched in the nucleus (Figure 2D) and indicates that AK085865 may play its biological function in the nucleus. Consistent with AK085865, which is defined as a non‐coding RNA, there is no open reading frame (ORF) longer than 200 bp in the sequence. The computational algorithm of the Coding Potential Assessment Tool (CPAT)^33^ predicts that AK085865 has very low coding potential, similar to the well‐known lncRNA HOTAIR (Figure 2E). Using an in vitro translation system, we found no evidence of AK085865 protein products (data not shown).

**FIGURE 3:**
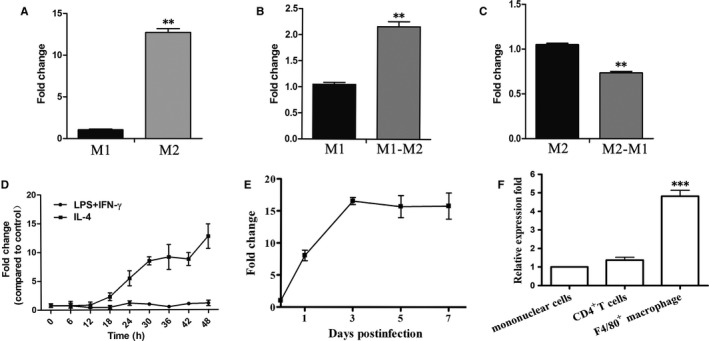
Expression pattern of lncRNA AK085865 during macrophage polarization. A, Levels of AK085865 in M1 and M2 were determined by RT‐qPCR *GAPDH* was used as an internal control. B‐C, M1 and M2 were then cultured in fresh media containing LPS and IFN‐γ or IL‐4 for 2 more days to induce the transition from M1 to M2 or vice versa. Levels of AK085865 were determined. D, The time course expression analysis of AK085865 during macrophage polarization. E, The time course expression analysis of AK085865 in heart tissues during acute myocarditis (n = 3/group). F, AK085865 expression was determined by RT‐qPCR in heart‐infiltrating cells from CVB3‐infected mice on day 7 (n = 6/group). Experiments were repeated three times in triplicate, and data were expressed as the means ± SD of three independent experiments. **P < .01; ***P < .001. Differences were determined by Student's *t* test

We next determined the expression change of AK085865 during macrophage polarization in vitro. We found that M2 macrophages represent high level of AK085865 than M1 macrophages (Figure 3A). To investigate whether AK085865 contributes to the plasticity of macrophage polarization, we attempted to culture M1 macrophages with IL‐4 and M2 macrophages with LPS plus IFN‐γ to convert one population into another. As shown in Figure 3B, M1‐to‐M2 conversion resulted in increased AK085865, whereas M2‐to‐M1 conversion led to decreased AK085865 expression. It is noteworthy that the stimulation of IL‐4 resulted in a significant increase in the expression of AK085865, which was significant at 18 hours and most obvious at 48 hours (Figure 3D). These findings suggest that AK085865 may be involved in macrophage polarization.

To determine whether the increase in AK085865 during M1‐to‐M2 conversion occurs in pathologic conditions where macrophage polarization plays important roles, we examined AK085865 levels in heart tissues from mice of VM. We found that the expression of AK085865 in the heart tissue of mice infected with CVB3 increased compared with that of PBS‐injected mice (Figure 3E). Since CD4^+^ T cells and macrophages are key mediators of VM, we then examined the expression of AK085865 in heart‐infiltrating mononuclear cells and found that AK085865 expression in macrophages was higher than in mononuclear cells (Figure 3F). Given the established role of M1 and M2 macrophages in VM, these data suggest that AK085865 may participate in VM through modulating macrophage polarization.

### 3.3 LncRNA AK085865 functions in macrophage polarization

**FIGURE 4:**
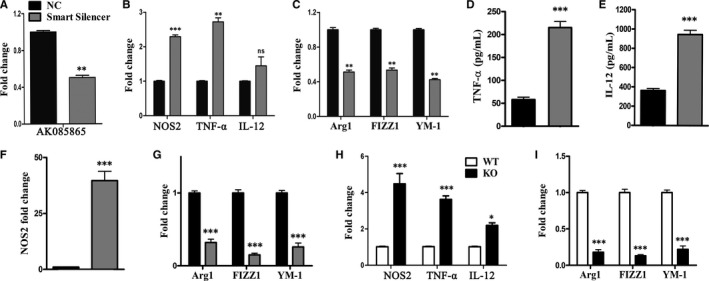
LncRNA AK085865 plays important roles in macrophage polarization. BMDMs were transfected with 100 nmol/L control siRNA or AK085865 Smart Silencer. Two days after transfection, the cells were treated with LPS (100 ng/mL) plus IFN‐ã (20 ng/mL) or IL‐4 (20 ng/mL) for 48 h. A, AK085865 expression was measured by RT‐qPCR *GAPDH* was used as internal control. B, Levels of NOS2, TNF‐α and IL‐12 were determined by RT‐qPCR. C, Levels of Arg1, YM‐1 and FIZZ1 were determined by RT‐qPCR. D‐G, Knock‐down of AK085865 promotes transition of M2 to the M1 phenotype and diminishes the expression of M2 phenotypes. M2 macrophages were transfected with 100 nmol/L control siRNA or AK085865 Smart Silencer. Two days after transfection, the cells were treated with LPS (100 ng/mL) plus IFN‐γ (20 ng/mL) or IL‐4 (20 ng/mL) for 48 h. Phenotypes of M1 and M2 were determined. Levels of TNF‐α and IL‐12 were determined by ELISA. Levels of NOS2, Arg1, YM‐1 and FIZZ1 were determined by RT‐qPCR. H‐I. Bone marrow cells were isolated by flushing the femurs of AK085865^−/−^ and WT mice, and BMDMs were prepared (n = 6‐8/group). Macrophage polarization was gained by dispensing the culture medium and culturing cells for an additional 48 h in DMEM supplemented with 10% FBS and LPS (100 ng/mL) plus IFN‐γ (20 ng/mL) or IL‐4 (20 ng/mL). Then, M1 and M2 macrophage‐specific markers were determined (H for M1, I for M2). Data were expressed as the means ± SD of three independent experiments. *P < .05; **P < .01; ***P < .001. NC, negative control; ns, no significance. Differences were determined by Student's *t* test (A‐I)

To clarify the function of AK085865 in macrophage polarization, AK085865 expression in bone marrow‐derived macrophages (BMDMs) was knocked down by AK085865 Smart Silencer (Figure 4A). Knock‐down of AK085865 in BMDMs promoted M1 phenotypical expression induced by LPS and IFN‐γ (Figure 4B) but diminished M2 marker expression induced by IL‐4 (Figure 4C). We next determined whether knock‐down of AK085865 in M2 macrophages achieves an effect similar to what had been observed in BMDMs that are transfected with AK085865 Smart Silencer. As shown in Figure 4D‐F, AK085865 knock‐down enhanced LPS‐ and IFN‐γ‐induced expression of TNF‐α, IL‐12 and NOS2. These data suggest that AK085865 plays an inhibitory role in M1 macrophage polarization. Furthermore, knock‐down of AK085865 diminished the IL‐4‐induced M2 macrophage phenotype, as shown by a decrease in IL‐4‐induced Arg1, FIZZ1 and YM‐1 in these cells (Figure 4G). These data further indicate that AK085865 functions in sustaining the M2 macrophage phenotype.

To further test our hypothesis that AK085865 regulates macrophage polarization, we generated mice lacking AK085865 (Figure S2) by deleting the complete 1922 bp genomic locus containing AK085865. The AK085865 KO mice were healthy and reproduced at the expected Mendelian frequency, without gender bias and any obvious developmental defects. To investigate whether AK085865 contributes to macrophage polarization, bone marrow cells from AK085865 KO and WT mice were isolated to prepare BMDMs. Macrophage polarization was induced by culturing cells under the condition of M1 or M2 polarization. As shown in Figure 4H, the expression of TNF‐a, IL‐12 and NOS2 in BMDMs of AK085865^−/−^ knockout mice was significantly increased after LPS and IFN‐γ treatment, while the expression of Arg1, FIZZ1 and YM‐1 in BMDMs of AK085865^−/−^ knockout mice induced by IL‐4 was significantly decreased (Figure 4I). These data confirmed that knockout of AK085865 inhibited the polarization of M2 macrophages and led to the development of macrophage polarization towards M1 phenotype.

### 3.4 LncRNA AK085865^−/−^ mice are more susceptible to CVB3‐induced VM

**FIGURE 5:**
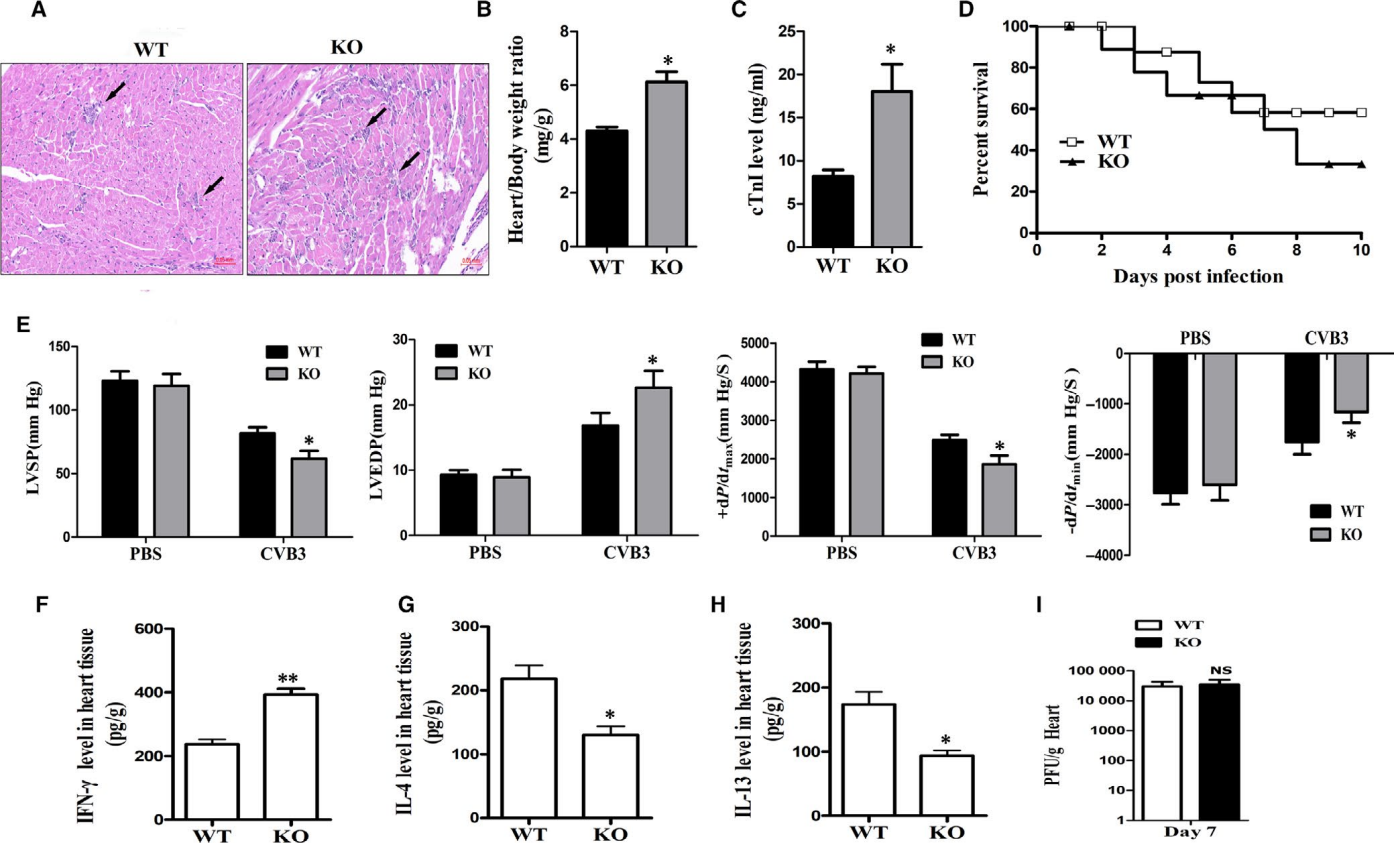
LncRNA AK085865^−/−^ mice are more susceptible to CVB3‐induced VM. AK085865^−/−^ and WT mice received 1 × 10^5^ PFU of CVB3 or PBS ip at day 0, and the heart samples were isolated at day 7. A, Heart sections of CVB3‐infected WT, and AK085865^−/−^ mice were stained with H&E, and the severity of myocarditis was assessed as the percentage of the heart section with inflammation compared with the overall size of the heart section, with the aid of a microscope eyepiece grid. Scale bar = 50 µm. B‐C. The parameters of the VM were evaluated including heart/bodyweight ratio (B) and levels of cTnI (C) on day 7 post‐infection. D, The survival rate of mice was observed until day 10 post‐infection. E, The left ventricular (LV) performance was assessed using a self‐made polyethylene catheter inserted via the right carotid artery. The pressure signal of LV was recorded continuously and stored in a computer to determine off‐line LV end‐diastolic pressure (LVEDP), LV systolic pressure (LVSP), maximum first derivative of LV pressure (d*P*/d*t*_max_) and minimum first derivative of LV pressure (−d*P*/d*t*_min_). F‐H. AK085865^−/−^ and WT mice received 1 × 10^5^ PFU of CVB3 ip on day 0, and protein levels of IFN‐γ, IL‐4 and IL‐13 in the homogenized heart tissue were determined by ELISA on day 7 post‐infection. I. Viral titres were measured using plaque assay. Experiments were repeated three times in triplicate with 10‐15 mice per group. Bar graph data are presented as mean ± SD; *P < .05 and **P < .01 as compared with WT mice. Differences were determined by Student's *t* test (B, C, E‐I) or Kaplan‐Meier Survival Analysis (D)

As macrophages are the key regulator of VM, we tried to determine the role of AK085865 in VM development. Compared with WT mice, AK085865^−/−^ mice showed a significant aggravation of VM exhibiting more foci of mononuclear inflammation (Figure 5A). In agreement with this observation, AK085865 deletion significantly increased the heart weight and also increased the cTnI levels in the serum (Figure 5B,C). Furthermore, AK085865 deletion significantly reduced the survival rate from about 60% to 40% after CVB3 infection (Figure 5D). In addition to decreasing the survival rate, AK085865 deletion aggravated cardiac function in the mice that survived following CVB3 infection (Figure 5E). AK085865^−/−^ mice survived to the observation time‐point had an increased left ventricular end‐diastolic pressure (LVEDP), a decreased left ventricular systolic pressure (LVSP), a decreased maximum first derivative of LV pressure (+d*P*/d*t*_max_) and a decreased minimum first derivative of LV pressure (−d*P*/d*t*_min_). In conclusion, silencing AK085865 aggravated mortality and cardiac dysfunction during VM.

To investigate the expression changes of cytokine at day 7 of VM, we used ELISA to detect the levels of inflammatory factors including IFN‐γ, IL‐4 and IL‐13 in heart tissue of AK085865^−/−^ mice and WT mice after heart homogenate. The results showed that CVB3 infection could induce IFN‐γ production both in WT and in AK085865^−/−^ mice (*P* = .00124) in heart tissues (Figure 5F), while the expression levels of IL‐4 and IL‐13 decreased (Figure 5G‐H), indicating that the level of anti‐inflammatory cytokines in heart tissues of AK085865^−/−^ mice infected with CVB3 was significantly decreased. Furthermore, we discovered that the viral replication in cardiac between all groups had no significant difference at the 7 day after CVB3 infection (Figure 5I).

The bias of CD4^+^ Th immune response greatly affects the severity of myocarditis. Therefore, the effect of AK085865 deletion on the activation phenotype of heart‐infiltrating CD4^+^ T cells in vivo was examined. Fluorescence‐activated cell sorting (FACS) analysis of heart‐infiltrating CD4^+^ T cells at day 7 demonstrated that compared to WT mice, AK085865^−/−^ VM mice showed increased activation as determined by increased proportions of CD4^+^ T cells expressing low levels of CD62L (Figure S3A). Moreover, BrdU incorporation assay further analysed the proliferation of CD4^+^ T cells in AK085865^−/−^ and WT VM mice. As shown in supplement Figure S3B, CD4^+^BrdU^+^ effector T cells from AK085865^−/−^ mice 7 days post‐infection were significantly increased compared to WT mice. Thus, heart‐infiltrating CD4^+^ T cells from AK085865^−/−^ VM mice exhibited more activated and had higher proliferative responses compared to WT VM mice.

### 3.5 Phenotypes of heart‐infiltrating macrophages of AK085865^−/−^ and WT mice

**FIGURE 6:**
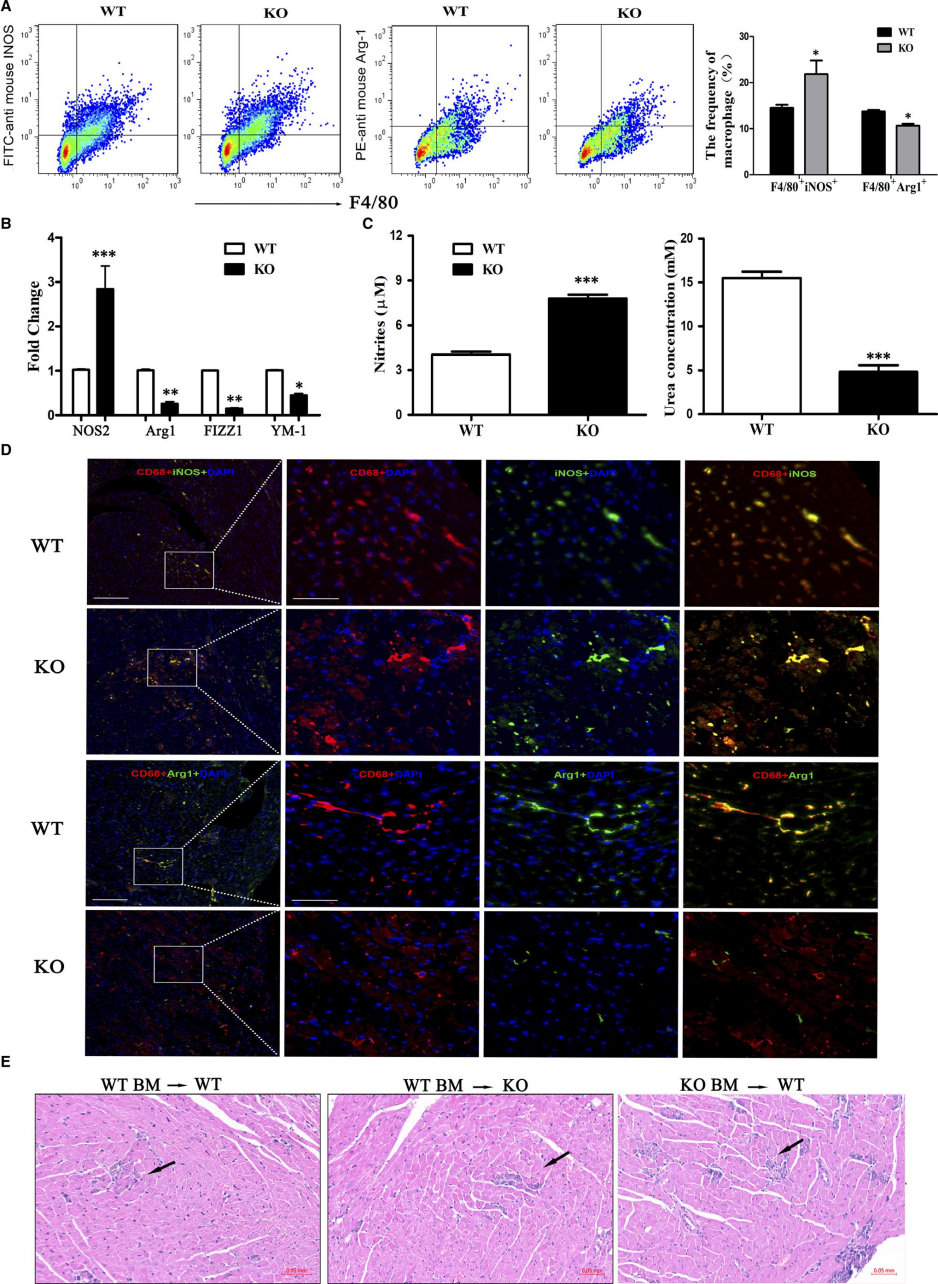
Phenotypes of heart‐infiltrating macrophages of AK085865^−/−^ and WT mice. A, Myocardial‐infiltrating leucocytes were isolated from the hearts after enzymatic digestion. The percentage of F4/80^+^iNOS^+^ or F4/80^+^Arg1^+^ cells were analysed by FACS. B, Phenotypic analysis of myocardial‐infiltrating macrophages in lncRNA AK085865^−/−^ and WT mice during CVB3‐induced VM. NOS2, Arg1, FIZZ1 and YM‐1 of sorted F4/80^+^ macrophages were determined by real‐time PCR. C, Tissue homogenate of myocardial‐infiltrating macrophages in lncRNA AK085865^−/−^ and WT mice during CVB3‐induced VM were prepared. NO production levels were observed using Griess activity assays and urea production using arginase activity assays. D, For colocalization analysis, sections were costained for CD68 (red, macrophage marker) and iNOS (green, M1 marker) or Arg1 (green, M2 marker). DAPI was used for nucleus staining (blue). Scale bar = 100 µm. E, Lethally irradiated WT or AK085865 KO mice were adoptively transferred with AK085865 KO or WT bone marrow, and then, the generated chimeric mice were subjected to CVB3‐induced myocarditis mouse model. Representative H&E staining of heart derived from indicated groups was shown, n = 5 per group. Scale bar: 50 μm. BM, bone marrow. Experiments were repeated three times in triplicate with 10‐15 mice per group. Bar graph data are presented as mean ± SD; *P < .05, **P < .01; ***P < .001 as compared with WT mice. Differences were determined by Student's *t* test

Accumulating data have indicated that macrophage polarization plays an essential role in the development of VM. Since our data have shown that AK085865^−/−^ mice are more prone to VM, we speculate that there may be macrophages with different functions in AK085865^−/−^ and WT mice. For this reason, we next determined the phenotype of heart‐infiltrating macrophages of AK085865^−/−^ and WT mice. FACS results showed that at the day 7 after CVB3‐induced VM, the proportion of F4/80^+^ macrophages expressing iNOS in the heart of AK085865^−/−^ VM mice was increased (Figure 6A). In addition, we observed a significant decrease in the proportion of F4/80^+^Arg1^+^ macrophages, which may suggest that M2 macrophages were decreased in the hearts of AK085865^−/−^ mice (Figure 6A). Consistently, the expressions of M2‐specific genes Arg1, FIZZ1 and YM‐1 in F4/80^+^ macrophages isolated from AK085865^−/−^ VM mice were decreased compared with those isolated from WT VM mice, while Nos2 expression was just opposite (Figure 6B). And the F4/80^+^ macrophages isolated from AK085865^−/−^ VM mice produced a large number of NO and showed low levels of arginase activity compared with those isolated from WT VM mice (Figure 6C).

Similar to the results of FACS analysis, colocalization demonstrated that iNOS signal in CD68^+^ macrophages derived from heart tissue of AK085865^−/−^ VM mice increased significantly, while the Arg1 signal was barely detected (Figure 6D). In summary, these results indicate that AK085865 plays a key role in the polarization of heart‐infiltrating macrophages during CVB3‐induced VM.

To explore the key role of AK085865 deletion in macrophages in the development of VM, we first constructed bone marrow (BM) chimeric mice using AK085865 KO and WT mice. Lethally irradiated AK085865 KO or WT mice were adoptively transferred to the BM of WT mice. The severity of myocardial inflammation in mice with two genotypes (WT BM‐KO and WT BM‐WT) was similar after CVB3 infection. However, compared with WT BM‐KO or WT BM‐WT mice, the WT recipient mice reconstituted with AK085865 KO mice (KO BM‐WT) showed significant VM aggravation (Figure 6E). These findings suggest that the deletion of AK085865 in macrophages is the main reason for the increased VM susceptibility induced by CVB3.

## 4 DISCUSSION

The mammalian genome encodes a regulatory RNAs that controls the expression of immune genes. Although the role of miRNAs in these processes has been well defined,^34^ little is known about the role of lncRNAs in the immune system. Abundant evidence indicates that lncRNAs are involved in the inflammation process.^35^, ^36^ In this paper, we investigated lncRNA expression profiles in ex vivo‐programmed M1 and M2 macrophages and identified an IL‐4‐induced up‐regulated lncRNA AK085865. Knock‐down of AK085865 inhibited the polarization of M2 macrophages and polarized macrophages to M1 phenotype in vitro and in vivo. As far as we know, AK085865 is the first lncRNA that has been defined to function as a regulator of macrophage polarization during CVB3‐induced VM.

During CVB3‐induced VM, macrophages were shown to be one of the predominant cell types and to display functional heterogeneity, with pro‐inflammatory M1 macrophages or anti‐inflammatory M2 macrophages. Research by our group and others suggests that excessive M1 macrophages may cause damage to the host after CVB3 infection, whereas M2 macrophages protect against CVB3‐induced VM. M1 macrophages express TNF‐α, iNOS, and IL‐12, and they induce a strong pro‐inflammatory reaction and contribute to VM. In contrast, M2 macrophages express Arg1 and IL‐10 instead of iNOS, depleting arginine stores so that NO is not produced, and, instead, produce polyamine and proline, which are important for cell differentiation and inflammatory response.^37^, ^38^

Using the approaches of gain of function and loss of function, we obtained an important understanding of AK085865 biological function at both the cellular and tissue levels. As expected, knock‐down of AK085865 in BMDMs promoted M1 phenotypical expression but diminished M2 marker expression. Our research also shows that AK085865 inhibits inflammation in vivo. In response to the challenge of CVB3, AK085865 knockout mice produced a high level of pro‐inflammatory cytokines. Furthermore, AK085865^−/−^ mice represented more susceptible to CVB3‐induced VM than their WT counterparts.

Rather than finding deviations in Th1/Th2/Tregs differentiation, we found differences in phenotypes of heart‐infiltrating macrophages when we compared AK085865^−/−^ and WT mice after CVB3 infection. Our work showed that macrophages of AK085865^−/−^ mice differentiated into M1 phenotype after CVB3 infection, which can effectively promote the development of VM. First, in AK085865^−/−^ mice, we observed higher level of myocardial IFN‐γ, which may be the cause of M1 polarization. Second, we found an increase in F4/80^+^ macrophages containing iNOS. These represent a typical activation subpopulation, whereas in the heart of AK085865^−/−^ mice, the number of F4/80^+^Arg‐1^+^ macrophages is reduced.

Although we are not sure that all these effects are due solely to the missing of AK085865 expression in the heart, our data provide convincing genetic evidence for in vivo functions of AK085865 in the development of VM. For further clarify the effect of AK085865 on specific cell types in some tissues during VM, selective genetic manipulation of cell types should be considered for future research. Moreover, the use of a simple M1/M2 model to describe macrophage polarization in vivo is debatable, and further studies should elucidate the exact macrophage phenotype and gene expression profile altered by the AK085865 ablation in myocardium in response to CVB3 infection. In summary, all these data indicate that AK085865 plays an essential role in acquiring and regulating M1/M2 phenotypes. Modulation of AK085865 level is very important for the development of a properly balanced immune response in VM.

In conclusion, we demonstrated in detailed molecular and genetic approaches that precise regulation of AK085865 expression in macrophages is necessary to control inflammation responses by modulating macrophage polarization. Therefore, AK085865, as an important node of the molecular circuit, can prevent the spontaneous activation of macrophages and has a potential effect on inflammation and immune diseases. The insights gained from this study further enhance our understanding of the physiological role of lncRNAs as a whole and the importance of these molecules in inflammatory heart disease.

## CONFLICT OF INTEREST

The authors confirm that there are no conflicts of interest.

## AUTHOR CONTRIBUTIONS

YYZ, XQL and XK designed the experiments and performed most of the experiments and analysed the data. MYZ and DGW assisted with hemodynamics analysis. YHL performed the experiments of histopathology. YYZ and KL conceived the project, analysed the data and wrote the manuscript.

## Supporting information

Supplementary Material

## Data Availability

The Gene Expression Omnibus (GEO) database accession number for the lncRNA profile data of M1 and M2 macrophages reported in this paper is http://www.ncbi.nlm.nih.gov/geo/query/acc.cgi?acc=GSE125510.
