# Supplementary material for: Long non‐coding RNA AK085865 ablation confers susceptibility to viral myocarditis by regulating macrophage polarization
Source: J Cell Mol Med. 2020 Mar 27;24(10):5542–54. doi: 10.1111/jcmm.15210 (PMC7214176; doi:10.1111/jcmm.15210)
Supplement: Supplementary file 1 — Supplementary Material [file JCMM-24-5542-s001.docx]

**Supplementary Figures and Figure Legends**

**
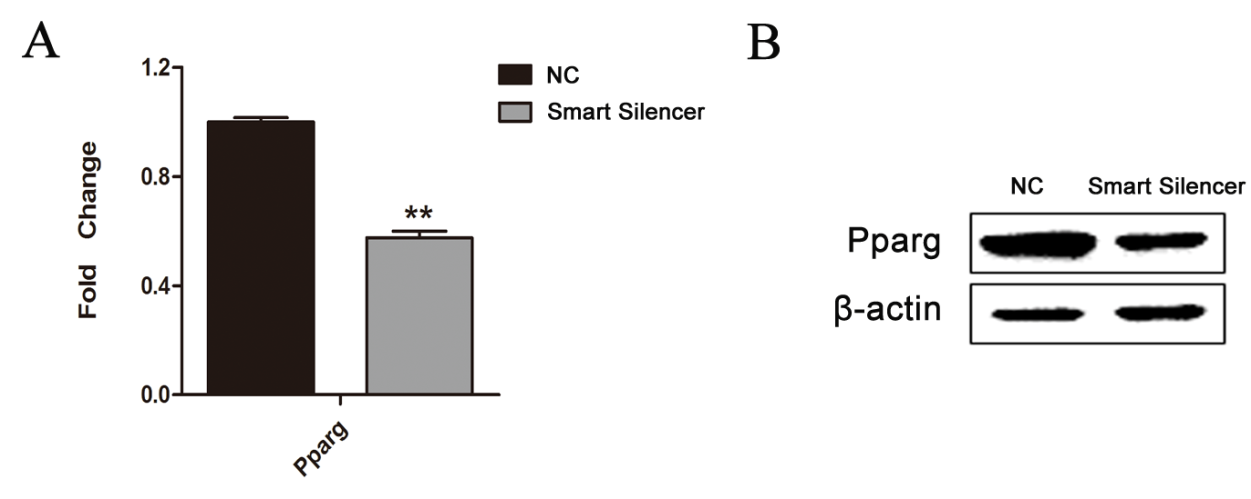
**

**Figure S1. LncRNA AK085865 regulates Pparg mRNA and protein expression *in vitro*.** **A.** Knockdown of AK085865 in BMDMs with lncRNA smart silencer. Pparg mRNA expression was assessed by RT-qPCR. **B.** Knockdown of AK085865 in BMDMs with lncRNA smart silencer. Pparg protein expression was assessed by western blot. Data represent the mean ± SD. Results are representative of at least three independent experiments with n ≥ 3. ***P* < 0.01.

**
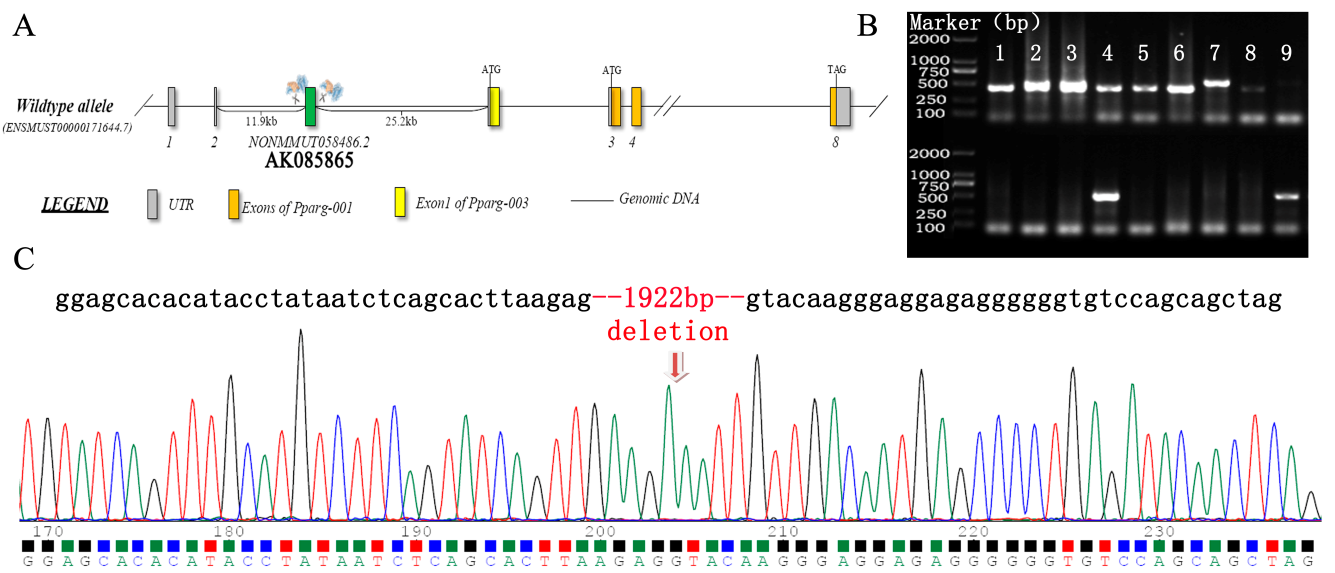
**

**Figure S2. Cas9/RNA-mediated gene targeting.** A. Schematic diagram of Cas9/RNA-mediated lncRNA AK085865 targeting. B. PCR amplification of the targeted fragment was performed using genomic DNA extracted from the tails of the founders as templates. Primers used were listed in Supplemental Table S1. C. The PCR products from founders were subjected to T-A cloning. Twenty colonies were randomly selected for DNA sequencing.


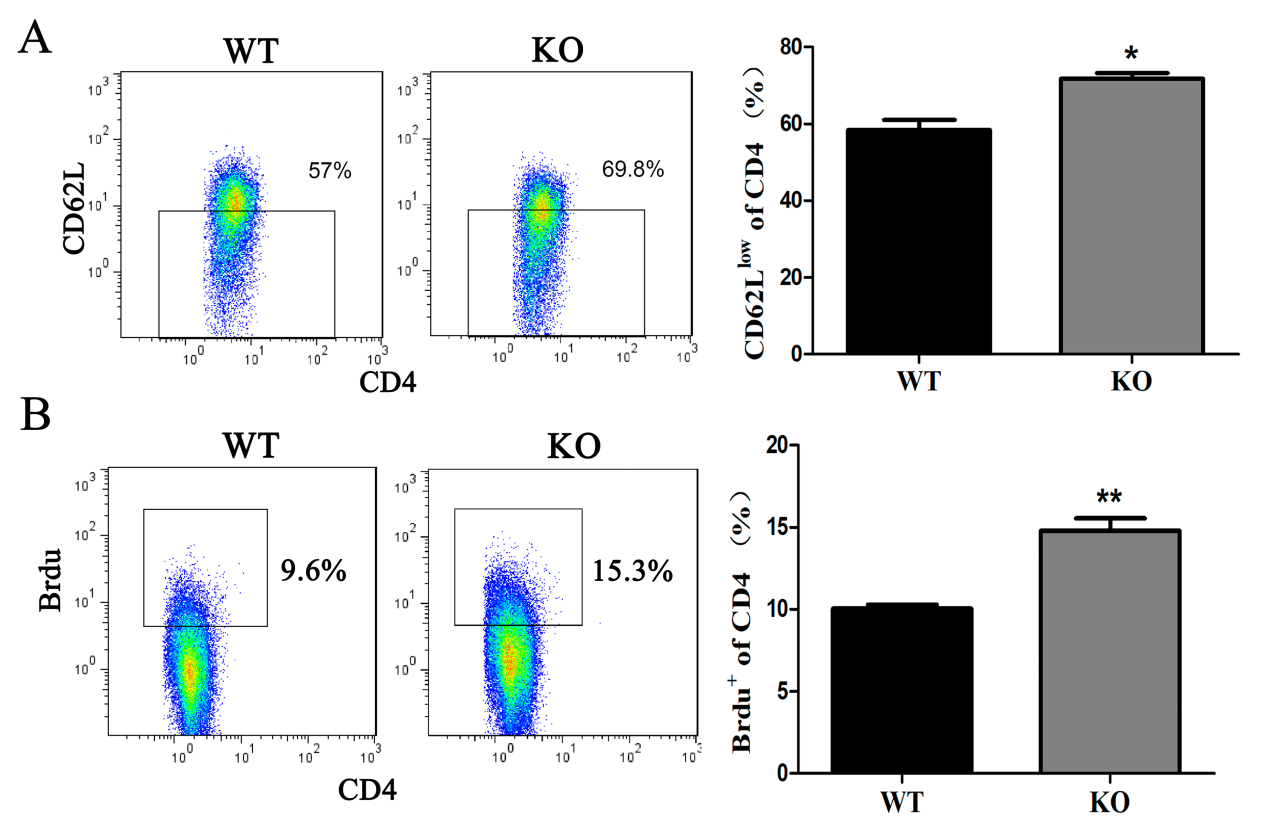


**Figure S3. lncRNA AK085865^-/-^ mice display decreased myocardial-infiltrating T cell activation during CVB3-induced VM.** AK085865^-/-^ and WT mice received 1×10^5^ PFU of CVB3 or PBS i.p. on day 0 and the heart samples were isolated on day 7. A. Myocardial-infiltrating leukocytes were isolated from the hearts after enzymatic digestion. Activated CD4^+^ T cells were determined by surface CD62L^low^ expression. B. 5-bromo-2-deoxyuridine (BrdU) was administered (0.8mg in 1ml PBS) 1 day before termination of mice. The proliferation of CD4^+^ T cells was determined by a BrdU incorporation assay *in vivo*. Experiments were repeated three times in triplicate with 10 mice per group. Bar graph data are presented as mean ± SD; **P* < 0.05 and ***P* < 0.01 as compared with WT mice. Differences were determined by Student’s t test.

**Supplement Tables**

**Supplement Table S1**. **Primer sequences used in real-time PCR are written in 5’- 3’ direction.**

| **Genes** | **Primer (5’- 3’)** |
| --- | --- |
| *AK048798* | GACGGATGAGGAATGGGTTC  TCCTGTGAAGTGTGGACTCT |
| *AK153212* | GTCAGAGCGGAAGTAAGGAC  TCTGCCTCCTGAGTAACACA |
| *AK085865* | ATGGAGTTTCAAGCTTGGCA  CCCAAGAACCAAAGAGCCAT |
| *AK083884* | TCACGCTATACAGGTGCAAC  AACAATGGCACTAGGTTGGG |
| *GAPDH* | GGTTGTCTCCTGCGACTTCA  TGGTCCAGGGTTTCTTACTCC |
| *Nos2* | ATCTTTGCCACCAAGATGGCCTGG  TTCCTGTGCTGTGCTACAGTTCCG |
| *TNF-α* | CCAGTGTGGGAAGCTGTCTT  AAGCAAAAGAGGAGGCAACA |
| *IL12* | GATGTCACCTGCCCAACTG  TGGTTTGATGATGTCCCTGA |
| *Arg1* | TGACTGAAGTAGACAAGCTGGGGAT  CGACATCAAAGCTCAGGTGAATCGG |
| *YM-1* | ATGAAGCATTGAATGGTCTGAAAG  TGAATATCTGACGGTTCTGAGGAG |
| *FIZZ1* | AGGTCAAGGAACTTCTTGCCAATCC  AAGCACACCCAGTAGCAGTCATCCC |
| *AK085865-WT* | TGTGAGGGTATATGACTGAC  GTCACACAAAGCAACTATGG |
| *AK085865-KO* | GTGTTGGAGGAGGAAGATGCTTC  CTAAGTCAACCACAGCACAGG |

**Supplement Table S2**. **Oligonucleotides sequences used in siRNA transfection are written.**

| **Genes** | **Target sequences** |
| --- | --- |
| *AK085865* Smart Silencer | GCCCTGTTGTAGTAACTTA (ASO)  ACCTTGGAAGAACTTACTT (ASO)  CCCTGTTGTAGTAACTTAA (ASO)  TTCAAGCTTGGCAGGTTATC (siRNA)  CAATTAGAGGCCACTGATTC (siRNA)  GAGACTAGAGGAAGTACATC (siRNA) |
